# Quantifying the learning curve for pulmonary thromboendarterectomy

**DOI:** 10.1186/s13019-017-0686-1

**Published:** 2017-12-28

**Authors:** Smita Sihag, Bao Le, Alison S. Witkin, Josanna M. Rodriguez-Lopez, Mauricio A. Villavicencio, Gus J. Vlahakes, Richard N. Channick, Cameron D. Wright

**Affiliations:** 1 Division of Thoracic Surgery, Massachusetts General Hospital, 55 Fruit Street, Founders 7, Boston, Massachusetts 02114 USA; 2 Division of Pulmonary and Critical Care Medicine, Massachusetts General Hospital, 55 Fruit Street, Boston, Massachusetts 02114 USA; 3 Division of Cardiac Surgery, Massachusetts General Hospital, 55 Fruit Street, Cox 6, Boston, Massachusetts 02114 USA; 4 Thoracic Surgery Service, Memorial Sloan Kettering Cancer Center, 12 75 York Avenue, C-881, New York, NY 10065 USA

**Keywords:** Pulmonary thromboendarterectomy, Outcomes, Learning curve

## Abstract

**Background:**

Pulmonary thromboendarterectomy (PTE) is an effective treatment for chronic thromboembolic pulmonary hypertension (CTEPH), but is a technically challenging operation for cardiothoracic surgeons. Starting a new program allows an opportunity to define a learning curve for PTE.

**Methods:**

A retrospective case review was performed of 134 consecutive PTEs performed from 1998 to 2016 at a single institution. Outcomes were compared using either a two-tailed t-test for continuous variables or a chi-squared test for categorical variables according to experience of the program by terciles (T).

**Results:**

The 30-day mortality was 3.7%. The mean length of hospital stay, length of ICU stay, and duration on a ventilator were 12.6 days, 4.6 days, and 2.0 days, respectively. The mean decrease in systolic pulmonary artery pressure (sPAP) was 41.3 mmHg. Patients with Jamieson type 2 disease had a greater change in mean sPAP than those with type 3 disease (*p* = 0.039). The mean cardiopulmonary bypass time was 180 min (T1–198 min, T3–159 min, *p* = <0.001), and the mean circulatory arrest time was 37 min (T1-44 min, T3-31 min, *p* < 0.001). Plotting circulatory arrest times as a running sum compared to the mean demonstrated 2 inflection points, the first at 22 cases and the second at 95 cases.

**Conclusions:**

PTE is a challenging procedure to learn, and good outcomes are a result of a multi-disciplinary effort to optimize case selection, operative performance, and postoperative care. Approximately 20 cases are needed to become proficient in PTE, and nearly 100 cases are required for more efficient clearing of obstructed pulmonary arteries.

## Background

Chronic thromboembolic pulmonary hypertension (CTEPH) is a relatively rare disease affecting less than 5% of patients subsequent to an acute pulmonary embolism [1]. It is characterized by pulmonary hypertension resulting from pulmonary vascular obstruction which leads to progressive right ventricular dysfunction. Riedel et al. reported that patients with mean pulmonary artery pressures greater than 30 mmHg have only a 30% 5-year survival, and in patients with mean PAPs greater than 50 mmHg, 5-year survival further decreases to as low as 10% [2]. Medical therapy is palliative and can mitigate symptoms in the short-term, while only surgical treatment via pulmonary thromboendarterectomy (PTE) offers the potential for cure [3].

PTE is a technically challenging operation that involves complete removal of organized fibrotic and thrombotic material from bilateral pulmonary arteries extending to segmental branches under intermittent deep hypothermic circulatory arrest [4]. The effectiveness of the operation is directly related to the location and accessibility of pulmonary arterial occlusive disease and the extent to which it is cleared by the surgeon [5]. It is performed predominantly at a few experienced centers across the country, and these centers have been able to demonstrate excellent outcomes in appropriately selected patients. Thus far, the group at University of California San Diego Medical Center (UCSD) has reported the largest experience with PTE with an overall mortality rate of 4.9% and a mean decrease in pulmonary artery systolic pressures of 28 mmHg correlating with a mean increase in cardiac output of 1.6 L/min. Reperfusion pulmonary edema was the most frequent postoperative complication at 10.9% [6]. In their more recent experience after completing 2700 cases, they reported a similarly substantial improvement in hemodynamics and cardiac function with an even lower mortality rate of 2.2% [7].

Our center initiated a program to surgically treat patients with CTEPH in 1998 as a product of a multi-disciplinary collaboration between experts in pulmonary hypertension and cardiothoracic surgery. Since then, we have performed a total of 134 cases, and we have averaged 31 cases per year over the past 3 years, thereby becoming the only high volume center in PTE in our region. All patients were selected by a multi-disciplinary team led by an experienced pulmonologist in CTEPH, and all PTEs were performed jointly by a cardiac and thoracic attending surgeon. Here, we report our experience with building a CTEPH program and define a learning curve for surgeons interested in achieving proficiency in PTE.

## Methods

All patients who underwent PTE from 1998 to 2016 at Massachusetts General Hospital in Boston, MA were retrospectively analyzed with the approval of the Partners Institutional Review Board Committee. A total of 134 patients were included in our study. Patients were selected for operation based on preoperative findings on pulmonary angiography, echocardiography, and nuclear ventilation-perfusion scans [8]. All cases were reviewed by a multi-disciplinary team of pulmonologists and cardiothoracic surgeons at our institution. PTE was performed jointly by a senior-level cardiac and thoracic attending surgeon in a manner similar to that described at UCSD [9]. A thoracic surgeon (CDW) performed or directly supervised all endarterectomies, and one of two cardiac surgeons assisted with cardiopulmonary bypass (GJV or MAV). Median sternotomy, cardiopulmonary bypass, aortic cross-clamping, hypothermia to 18 degrees °C, and periods of circulatory arrest of up to 20 min for each side were all essential components of operative conduct for optimal exposure and clearance of pulmonary vasculature. Additional cardiac procedures were performed during the re-warming period if indicated. Preoperative hemodynamics were assessed during right heart catheterization, while postoperative hemodynamics were monitored via Swan-Ganz catheter (Edwards Life Sciences, Irvine, CA) in the operating room and intensive care unit. Of note, pulmonary capillary wedge pressures (PCWP) were not measured routinely in our intensive care unit postoperatively. All surgical, hemodynamic, and 30-day postoperative outcomes were recorded in our database.

For data review, patients were divided into 3 evenly distributed groups or terciles by case number. The first tercile (T1) (*n* = 44) spanned from October 1998 to September 2012 and represented a more sporadic experience that began ramping up in 2010. The second tercile (T2) (*n* = 45) extended from November 2012 to February 2015, and the third tercile (T3) (*n* = 45) from February 2015 to August 2016. Statistical comparisons between T1 and T3 were carried out using a two-tailed unpaired student’s t test for continuous variables, and a Chi-squared test for categorical variables. Significance was set at a threshold of *p* < 0.05. We utilized STATA statistical software package, release 14 (StataCorp LP, College Station, TX) for all analyses.

To compute a learning curve, we plotted the cumulative running sum (CUSUM) of the difference of each value from the mean deep hypothermic circulatory arrest time across 134 consecutive cases, as this parameter improved dramatically with increasing surgeon experience. This methodology has been previously validated in the surgical literature for defining a learning curve for complex procedures [10, 11].

## Results

We included all consecutive patients who underwent PTE since the inception of our institution’s CTEPH program in 1998 (*n* = 134) and divided them into terciles (T1 – T3) to evaluate surgical, hemodynamic, and postoperative outcomes over time as our experience increased. Preoperative characteristics of all patients in our study are summarized in Table 1. Patients had an average age of 54 years, and 60% were male. The degree of pulmonary hypertension in these patients was severe with an average systolic pulmonary artery pressure (sPAP) of 78 mmHg and diastolic pulmonary artery pressure (dPAP) of 28 mmHg. Mean pulmonary vascular resistance (PVR) was calculated at 639 dynes-sec-cm^−5^. Preoperatively, 87.3% of patients were classified as New York Heart Association (NYHA) Heart Failure Class II or III, and nearly all patients had either Jamieson type 2 or 3 disease (none had type 4 disease) [5]. These preoperative parameters did not vary significantly by tercile.

**Table 1:** Preoperative patient characteristics

	Overall	Tercile (T)			*P* value
Variable	(*n* = 134)	T1 (*n* = 44)	T2 (*n* = 45)	T3 (*n* = 45)	T1 vs. T3
Age (y)	54 ± 15	54 ± 14	54 ± 14	53 ± 16	0.757
Male Sex (%)	81 (60.4)	22 (50.0)	32 (71.1)	27 (60.0)	0.343
PAP (mm Hg)					
Systolic	78 ± 20	77 ± 19	78 ± 21	78 ± 20	0.778
Diastolic	28 ± 9	26 ± 10	30 ± 8	27 ± 9	0.882
PVR (dynes-sec-cm^−5^)	639 ± 373	695 ± 442	618 ± 371	602 ± 292	0.243
CO (L/min)	4.7 ± 1.5	4.5 ± 1.5	4.8 ± 1.4	4.7 ± 1.5	0.531
DLCO (% predicted)	64 ± 19	66 ± 17	67 ± 20	56 ± 18	0.011
NYHA class					
I	1 (0.7)	0 (0)	1 (2.2)	0 (0)	0.218
II	43 (32.1)	17 (38.6)	14 (31.1)	12 (26.7)	
III	74 (55.2)	21 (47.7)	26 (57.8)	27 (60.0)	
IV	15 (11.2)	6 (13.6)	3 (6.7)	6 (13.3)	
Jamieson classification					
Type 1	1 (0.7)	0 (0)	1 (2.2)	0 (0)	0.226
Type 2	64 (47.8)	24 (54.5)	20 (44.4)	20 (44.4)	
Type 3	61 (45.5)	20 (45.5)	17 (37.8)	24 (53.3)	
Type 4	0 (0)	0 (0)	0 (0)	0 (0)	

Data are shown as mean ± standard deviation or numbers (percentages)

*PAP* pulmonary artery pressure, *PVR* pulmonary vascular resistance, *CO* cardiac output, *DLCO* diffusing capacity of carbon monoxide, *NYHA* New York Heart Association

Postoperatively, we observed a mean decrease in sPAP of 41 mmHg and a mean decrease in right atrial pressure (RAP) of 6 mmHg overall (Table 2). The mPAP decreased from 49 mmHg to 22 mmHg overall, and there was no difference in the number of patients with residual pulmonary hypertension (mPAP >30 mmHg) across terciles. These favorable hemodynamic results following PTE were achieved consistently across all 3 terciles of patients, and therefore increased surgeon experience did not appear to influence the degree of improvement in pulmonary artery pressures. Rather, patients with Jamieson type 2 disease had a significantly greater mean decrease in sPAP than patients with Jamieson type 3 disease (45 vs. 37, *p* = 0.039), further validating that anatomic and pathological classification of thromboembolic disease in the pulmonary arteries is a major predictor of outcome after PTE [5].

**Table 2:** Comparison of perioperative hemodynamic and surgical parameters

	Overall	Tercile (T)			*P* value
Variable	(*n* = 134)	T1 (*n* = 44)	T2 (*n* = 45)	T3 (*n* = 45)	T1 vs. T3
Postoperative					
sPAP (mm Hg)	37 ± 13	38 ± 13	35 ± 13	40 ± 14	0.468
mPAP (mm Hg)	22 ± 7	21 ± 6	20 ± 7	23 ± 8	0.183
RAP (mm Hg)	5 ± 4	5 ± 3	5 ± 4	5 ± 3	1.000
Mean Δ sPAP (mm Hg)	41 ± 21	40 ± 19	43 ± 22	41 ± 21	0.908
Mean Δ mPAP (mm Hg)	24 ± 13	25 ± 11	24 ± 13	24 ± 14	0.570
Mean Δ RAP (mm Hg)	6 ± 8	7 ± 11	6 ± 6	5 ± 6	0.287
CPB time (min)	180 ± 41	198 ± 42	182 ± 42	159 ± 28	<0.001
Aortic cross-clamp time (min)	132 ± 31	140 ± 35	134 ± 28	123 ± 28	0.018
DHCA time (min)	37 ± 15	44 ± 17	35 ± 13	31 ± 10	<0.001

Data are shown as mean ± standard deviation

*sPAP* systolic pulmonary artery pressure, *mPAP* mean pulmonary artery pressure, *RAP* right atrial pressure, *CPB* cardiopulmonary bypass, *DHCA* deep hypothermic circulatory arrest

Similarly, mortality and other postoperative outcomes remained relatively consistent across our experience (Table 3). The 30-day mortality rate following PTE was 3.7% overall, which is on par with other high volume centers [12, 13]. In the postoperative setting, 44.4% of patients experienced at least one complication, 20.8% of patients experienced a major complication, and 16.4% of patients experienced at least one pulmonary complication (Table 3). By far, the most common complication was atrial fibrillation at 29.7%, and major complications included acute respiratory distress syndrome, pneumonia, or respiratory failure requiring re-intubation, sepsis, and return to the operating room for any reason. Though the rate of major complications trended downward across terciles, this was not statistically significant. A total of 4 patients in our series had massive hemoptysis postoperatively, and there was no correlation with surgeon experience. The average length of hospital stay was 12.6 days, with an average of 4.6 days in the intensive care unit and 2.0 days on the ventilator. After undergoing PTE, 74.6% of patients had marked relief of their symptoms and were reclassified as NYHA class I from class II-IV, and 65% of patients in T1 were NYHA class I in comparison to 80% in T3 (*p* = 0.15).

**Table 3:** Comparison of postoperative outcomes

	Overall	Tercile			*P* value
Variable	(*n* = 134)	T1 (*n* = 44)	T2 (*n* = 45)	T3 (*n* = 45)	T1 vs. T3
30-day mortality	5 (3.7)	0 (0)	3 (6.6)	2 (4.4)	0.159
At least one complication	72 (44.4)	28 (63.6)	17 (37.8)	27 (60.0)	0.724
At least one pulmonary complication	19 (14.8)	5 (11.4)	7 (15.6)	7 (15.6)	0.563
At least one major complication	26 (20.3)	11 (25.0)	9 (20.0)	6 (13.3)	0.162
Residual mPAP >30 mmHg	11 (8.2)	4 (9.1)	4 (8.9)	3 (6.7)	0.671
30-day readmission rate	7 (5.2)	3 (6.8)	0 (0)	4 (8.9)	0.713
Length of hospital stay (d)	12.6 ± 9.0	12.8 ± 7.8	13.0 ± 11.7	11.8 ± 6.7	0.517
Length of ICU stay (d)	4.6 ± 4.7	4.5 ± 3.4	4.7 ± 5.6	4.6 ± 4.8	0.910
Duration on ventilator (d)	2.0 ± 3.0	1.6 ± 1.5	2.6 ± 4.5	1.8 ± 2.2	0.618

Data are shown as mean ± standard deviation or numbers (percentages)

*ICU* intensive care unit, *mPAP* mean pulmonary artery pressure

**Fig. 1:**
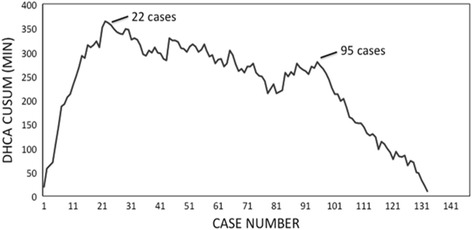
CUSUM plot depicts learning curve for PTE. Cumulative running sum (CUSUM) of the difference in DHCA (deep hypothermic circulatory arrest) time from the mean value is plotted on the vertical axis vs. case number on the horizontal axis. Two inflection points are seen at 22 and 95 cases

We did, however, detect a significant improvement in operative parameters with increased surgeon experience. Total cardiopulmonary bypass, aortic cross-clamp, and deep hypothermic circulatory arrest times steadily shortened from T1 to T3, and these differences were statistically significant (Table 2). Total cardiopulmonary bypass time decreased from a mean of 198 min to 159 min (*p* < 0.001) from T1 to T3, and total deep hypothermic circulatory arrest time decreased from a mean of 44 min to 31 min (*p* < 0.001). In order to further illustrate a learning curve of PTE, Fig. 1 depicts a CUSUM plot of deep hypothermic circulatory arrest times over 134 cases as compared to the mean value. Interestingly, two inflection points are seen in this curve at 22 and 95 cases. The first inflection point at 22 cases suggests a threshold of transitioning from novice to intermediate with PTE, as arrest times begin to trend downward thereafter. After the second inflection point at 95 cases, arrest times plummet even more steeply, indicating a threshold of attaining advanced expertise in clearing obstructed pulmonary arteries.

## Discussion

All complex procedures are associated with a learning curve for surgeons, and PTE is among one of the more technically demanding operations in cardiothoracic surgery. It is undoubtedly a challenging procedure to learn, and our experience demonstrates that favorable outcomes can be achieved in the setting of a dedicated multi-disciplinary team of pulmonologists, surgeons, and intensivists working in concert to select appropriate patients, improve operative efficiency, and provide meticulous postoperative care. Accurate diagnosis of CTEPH and recognition of patients with disease amenable to surgical intervention are the first steps, and having the benefit of an expert pulmonologist in CTEPH as part of our team has greatly facilitated evaluation and subsequent referral of excellent surgical candidates. The other key components included the involvement of two senior-level cardiothoracic surgeons on each case, and 24-h coverage of our cardiothoracic surgery ICU by a certified intensivist. Even early in our program’s existence, we were able to obtain adequate clearance of obstructed pulmonary arteries with significant postoperative hemodynamic improvements, despite much longer operative and circulatory arrest times. However, overall morbidity and mortality were not adversely affected during the initial phases of building our program, as there were no early deaths in our first 44 cases.

As we would expect, our learning curve suggests that operative efficiency is greatly enhanced with increasing case numbers. According to Fig. 1, approximately 20 cases are needed for a surgeon to become proficient at reliably recognizing and dissecting the thromboendarterectomy plane during circulatory arrest, while nearly 100 cases are needed to demonstrate mastery. To our knowledge, no other high volume center has described a learning curve for this procedure. The main limitation of our study is the fact that we have described the learning curve of a single team of providers at a single institution based on a retrospective analysis. We believe that the fundamental tenets that allowed us to successfully build our program, however, can be generalized and may prove useful to other programs early on in their trajectory. Surgical treatment of patients with CTEPH remains largely focused at regional centers of excellence at this time. As of 2013, only approximately 30 centers worldwide offered PTE surgery, and over half of cases were performed at the highest volume program at UCSD [14]. As CTEPH appears to be gaining prevalence in the U.S. and the limits of surgically curable disease are expanding [15–17], the need for more widespread access to PTE surgery is surely to arise in the future.

## Conclusions

In summary, our experience offers other centers which aspire to build a multi-disciplinary program to treat CTEPH a road-map on how to achieve this goal. We conclude that adopting a team-based approach with an emphasis on proper patient selection and adequate surgical clearance of obstructed pulmonary arteries can lead to favorable outcomes from the outset. Operative expediency is largely a function of case volume, and may be less critical with respect to influencing surgical and hemodynamic outcomes.

## Abbreviations

CTEPH: Chronic thromboembolic pulmonary hypertension
CUSUM: Cumulative running sum
DHCA: Deep hypothermic circulatory arrest
dPAP: Diastolic pulmonary artery pressure
ICU: Intensive care unit
mPAP: Mean pulmonary artery pressure
NYHA: New York Heart Association
PAP: Pulmonary artery pressure
PCWP: Pulmonary capillary wedge pressure
PTE: Pulmonary thromboendarterectomy
PVR: Pulmonary vascular resistance
RAP: Right atrial pressure
sPAP: Systolic pulmonary artery pressure
T: Tercile
